# Supplementary material for: Trandolapril Attenuates Pro-Arrhythmic Downregulation of Cx43 and Cx40 in Atria of Volume Overloaded Hypertensive and Normotensive Rats
Source: Biomolecules. 2025 Oct 15;15(10):1457. doi: 10.3390/biom15101457 (PMC12562952; doi:10.3390/biom15101457)
Supplement: Supplementary file 1 [file biomolecules-15-01457-s001.zip › biomolecules-3821462-supplementary.docx]

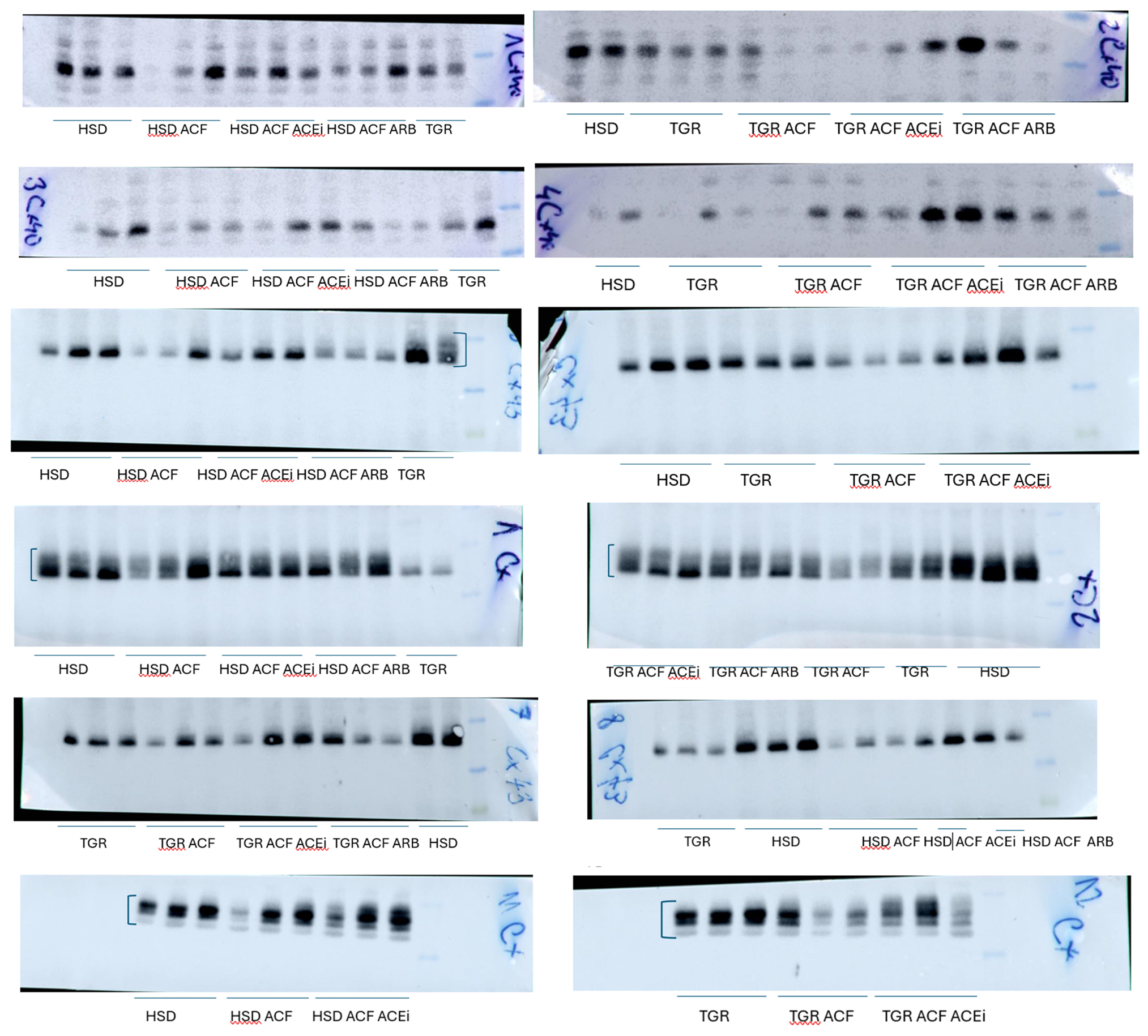


**Figure S1.** Protein levels of Cx43 and Cx40 normalized to GAPDH assessed by Western blot analysis


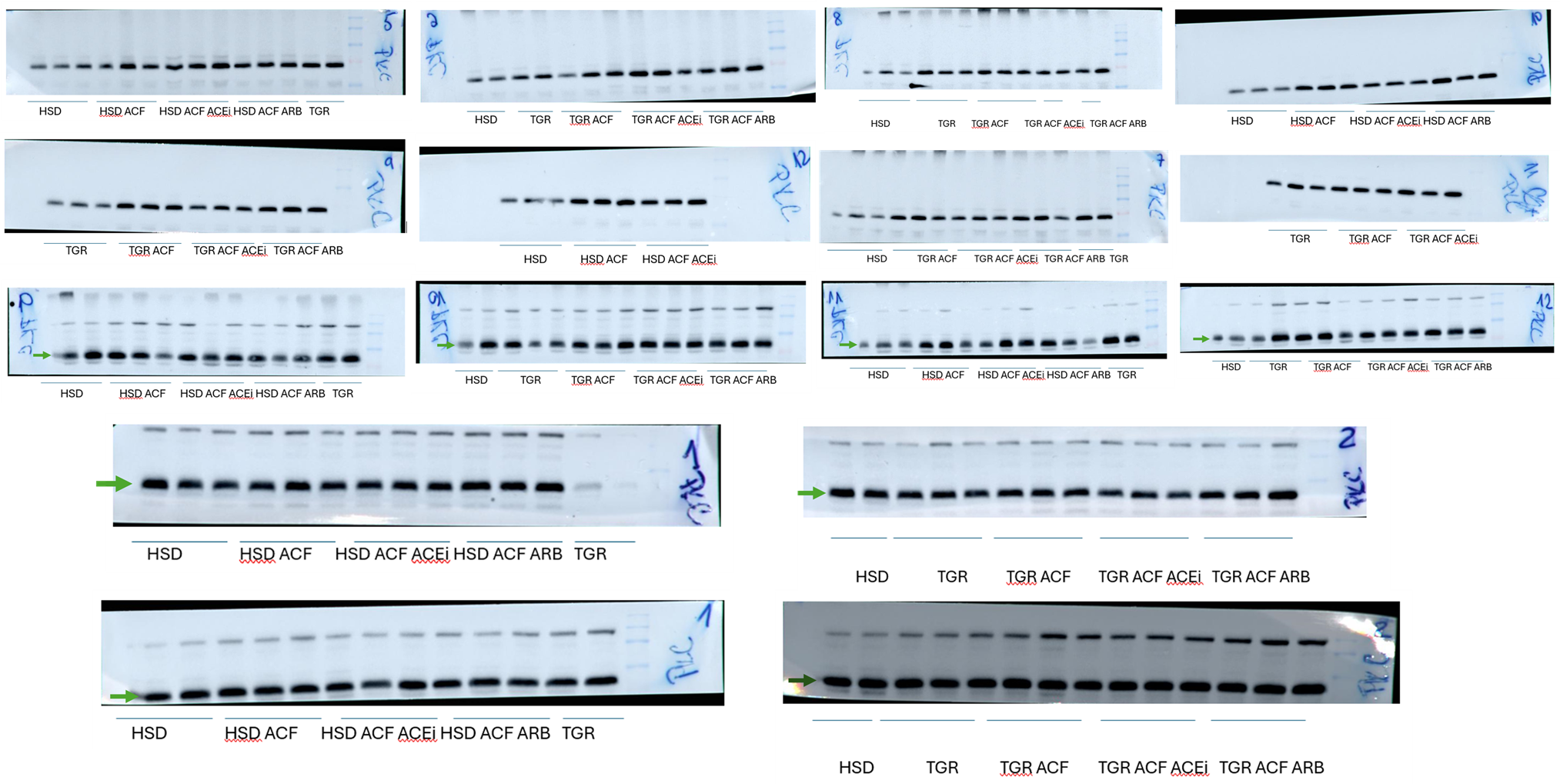


**Figure S2.** Protein levels of PKCε and PKCδ normalized to GAPDH assessed by Western blot analysis.


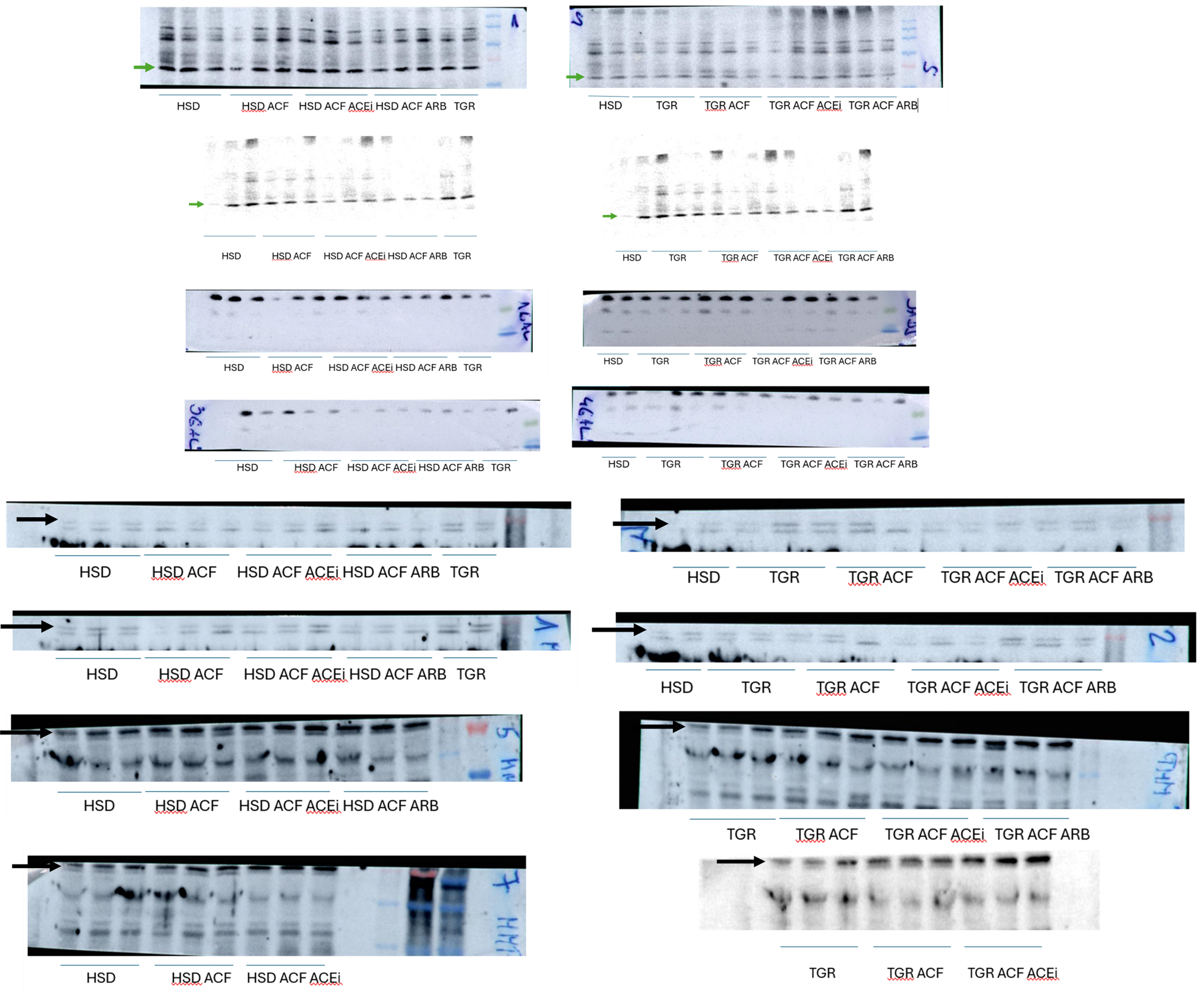


**Figure S3.** Protein levels of MMP-2, Galectin-3, and ADAMTS normalized to GAPDH assessed by Western blot analysis.


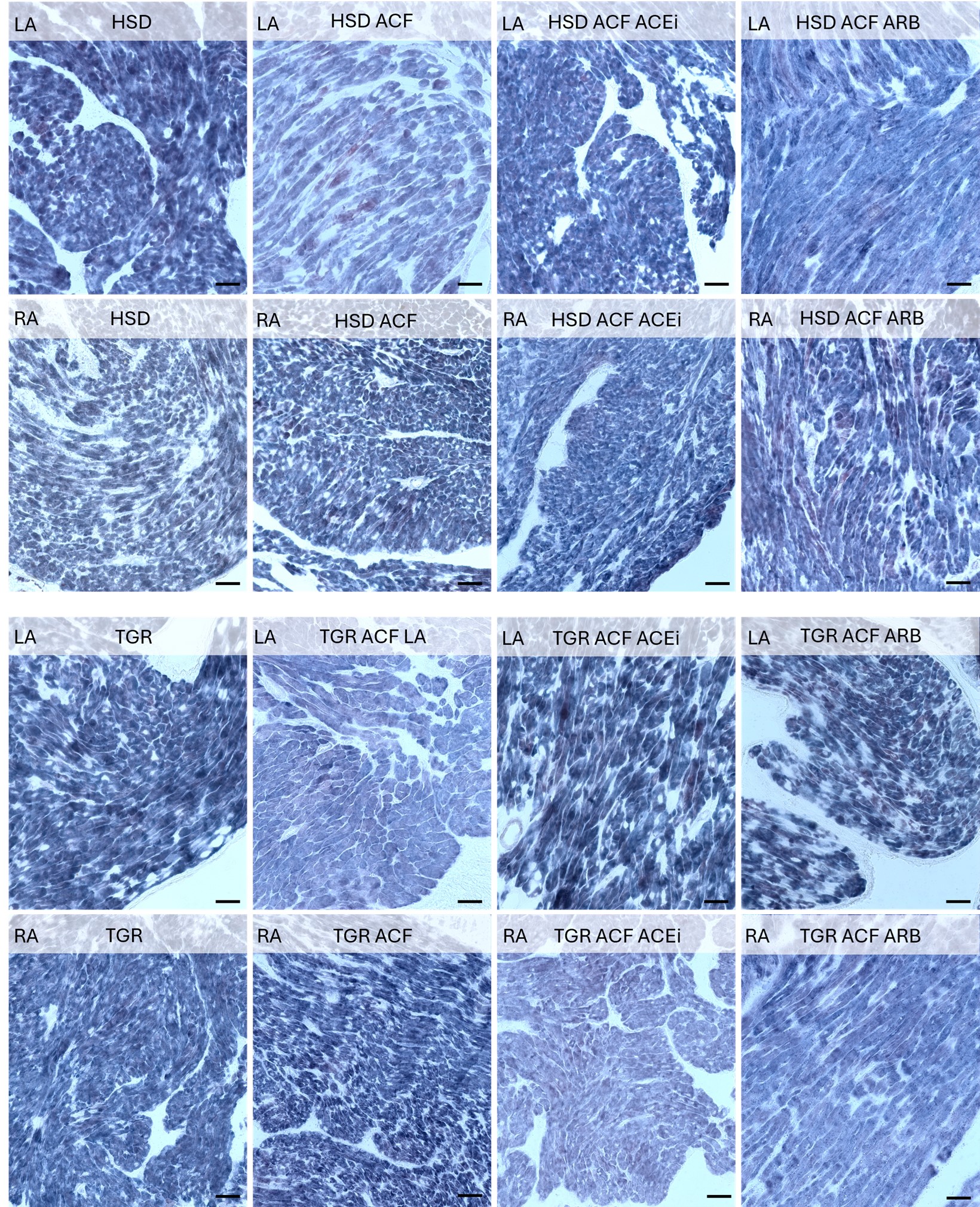


**Figure S4.** Microscopic images of glycogen phosphorylase activity in the left (LA) and right atria (RA) of normotensive (HSD) and hypertensive (TGR) rats in response to volume overload (ACF) and treatment with trandolapril (ACEi) or losartan (ARB). Glycogen phosphorylase is an enzyme essential for breaking down glycogen into glucose, thereby providing energy during periods of increased metabolic demand. The strong intensity of blue staining, indicating enzyme activity, did not suggest the presence of severe ischemia under volume overload conditions. HSD—Hannover Sprague Dawley rats; TGR—Ren-2 transgenic rats; ACF—aortocaval fistula, surgical model of volume overload; ACEi—treatment with the angiotensin-converting enzyme inhibitor, trandolapril; ARB—treatment with an angiotensin II type 1 (AT_1_) receptor blocker, losartan. Scale bar: 100 μm.

**
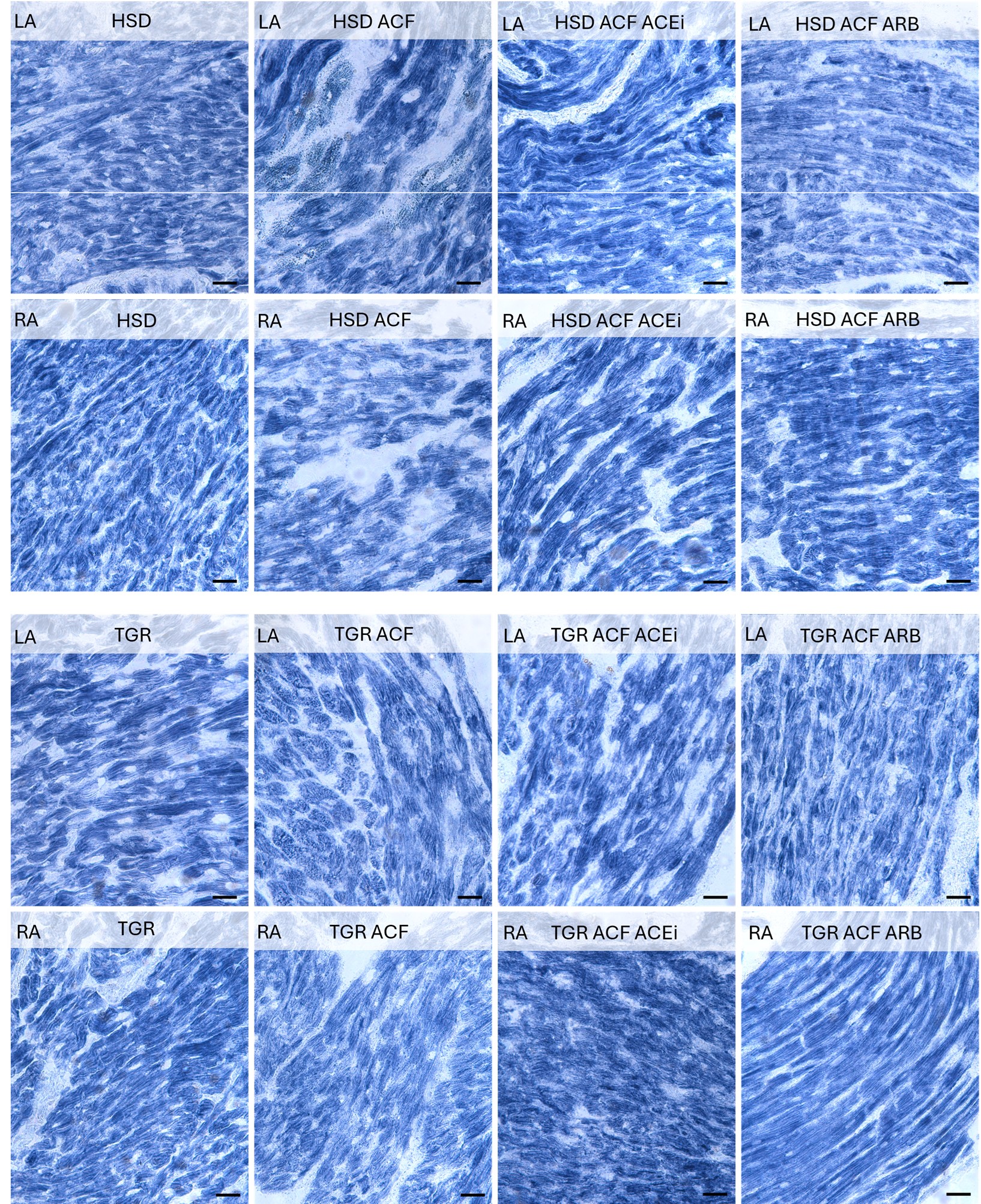
Figure S5.** Microscopic images of mitochondrial succinate dehydrogenase activity in the left (LA) and right atria (RA) of normotensive (HSD) and hypertensive (TGR) rats in response to volume overload (ACF) and treatment with trandolapril (ACEi) or losartan (ARB). The strong intensity of blue staining, corresponding to enzyme activity, indicates preserved mitochondrial function in response to volume overload or treatment, regardless of rat strain. . HSD—Hannover Sprague Dawley rats; TGR—Ren-2 transgenic rats; ACF—aortocaval fistula, surgical model of volume overload; ACEi—treatment with the angiotensin-converting enzyme inhibitor, trandolapril; ARB—treatment with an angiotensin II type 1 (AT_1_) receptor blocker, losartan. Scale bar: 100 μm.


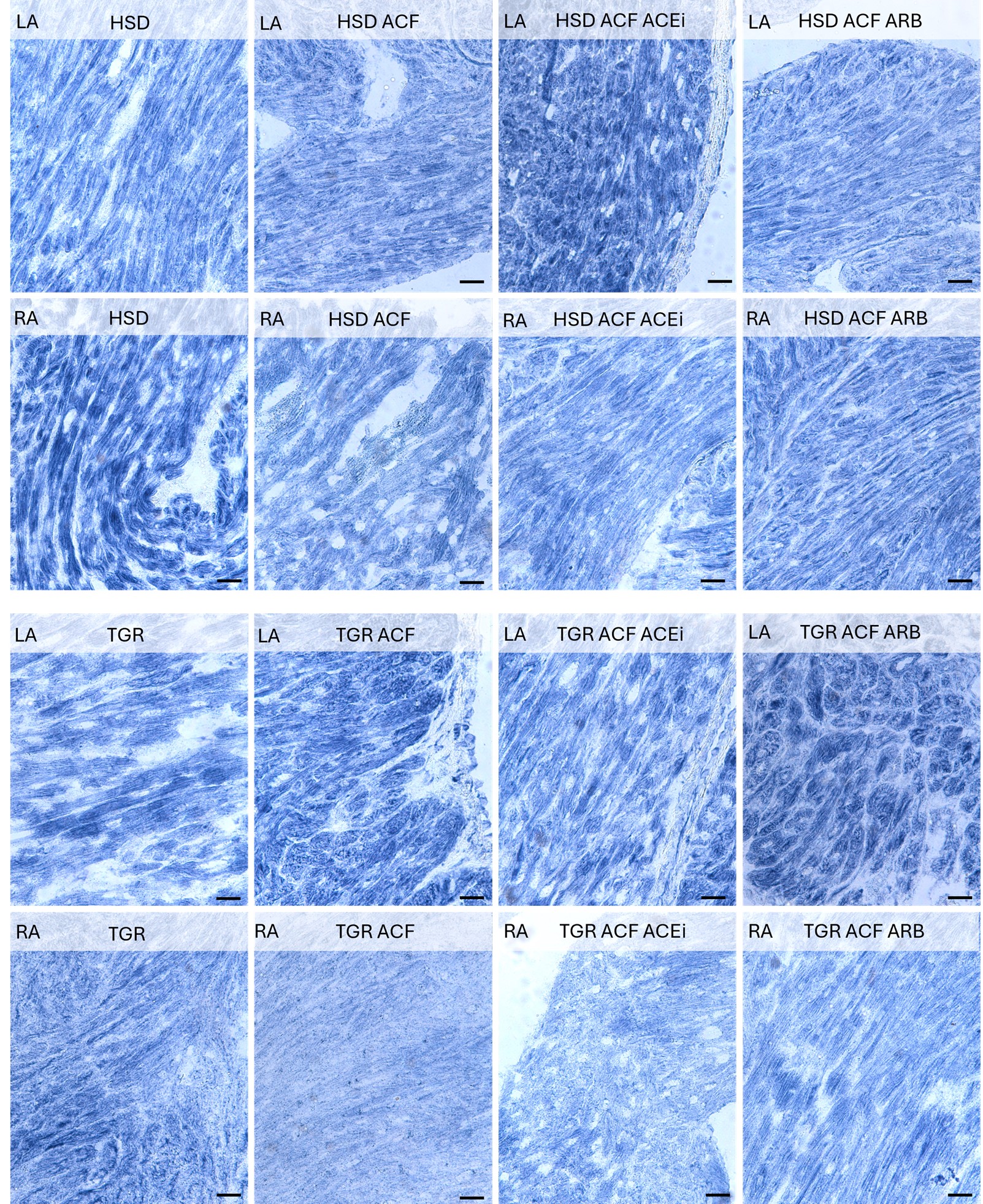


**Figure S6.** Microscopic images of mitochondrial beta-hydroxybutyrate dehydrogenase activity in left (LA) and right heart atria (RA) of normotensive (HSD) and hypertensive (TGR) rats were detected in response to volume overload (ACF) and to treatment with trandolapril (ACEi) and losartan (ARB). Strong intensity of blue staining corresponding to the enzyme activity indicates preservation of mitochondrial function in response to volume overload or treatment regardless rat strain. HSD—Hannover Sprague Dawley rats; TGR—Ren-2 transgenic rats; ACF—aortocaval fistula, surgical model of volume overload; ACEi—treatment with the angiotensin-converting enzyme inhibitor, trandolapril; ARB—treatment with an angiotensin II type 1 (AT_1_) receptor blocker, losartan. Scale bar indicates 100 μm.
